# *sym-2* loss-of-function causes glutamatergic neurodegeneration after oxidative stress

**DOI:** 10.17912/micropub.biology.000363

**Published:** 2021-02-03

**Authors:** Veronica H. Ryan, Anne C. Hart

**Affiliations:** 1 Neuroscience Graduate Program, Brown University, Providence, RI 02912, USA; 2 Department of Neuroscience, Brown University, Providence, RI 02912, USA

## Abstract

Although some RNA-binding proteins are known to contribute to neurodegeneration, the genetic interaction between the genes encoding these proteins is unclear. Here, we examine the interaction between *sym-2*, the gene encoding an ortholog of hnRNPF and hnRNPH, and *hrpa-1*, the ortholog of of the gene encoding hnRNPA2, which when mutated causes multisystem proteinopathy. We find that after 22 hours, but not 4 hours, of paraquat-induced oxidative stress, *sym-2(mn617)* has a mild glutamatergic neurodegeneration phenotype. Interestingly, this defect is rescued by expression of chimeric WT *hrpa-1*, but not mutant. Thus, we identify a curious genetic interaction between *sym-2* and *hrpa-1*.

**Figure 1. sym-2(mn617) neurodegeneration is rescued by expression of hrpa-1HsLCWT:**
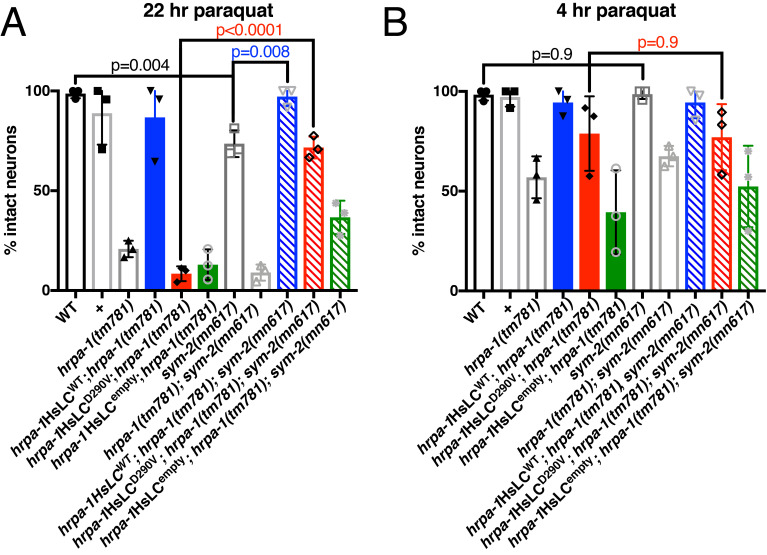
**A)** After exposure to 22 hours of paraquat-induced oxidative stress, *sym-2(mn617)* causes a modest dye filling defect in the phasmid neurons as compared to N2 animals, indicative of glutamatergic neurodegeneration. *hrpa-1*HsLC^WT^, but not D290V, empty, or *hrpa-1(tm781)*, rescues this defect. One-way ANOVA F=51.28, p<0.0001 with p-values corrected for multiple comparisons of 0.0753 for WT v. *sym-2(mn617)*, 0.1082 for *sym-2(mn617)* v. *hrpa-1*HsLC^WT^; *hrpa-1(tm781)*; *sym-2(mn617)*, and <0.0001 for *hrpa-1*HsLC^D290V^; *hrpa-1(tm781)* v. *hrpa-1*HsLC^D290V^; *hrpa-1(tm781)*; *sym-2(mn617)*. **B)** However, after exposure to only 4 hours of paraquat-induced oxidative stress, *sym-2(mn617)* does not cause a dye filling defect. One-way ANOVA F=8.353, p<0.0001 with p-values corrected for multiple comparisons of >0.9999 each for WT v. *sym-2(mn617)*, *sym-2(mn617)* v. *hrpa-1*HsLC^WT^; *hrpa-1(tm781)*; *sym-2(mn617)*, and *hrpa-1*HsLC^D290V^; *hrpa-1(tm781)* v. *hrpa-1*HsLC^D290V^; *hrpa-1(tm781)*; *sym-2(mn617)*. WT are N2 animals; + are wild type progeny from *tmC25[tmIs1241]/+* mothers. N=7-12 animals/genotype/trial, 3 trials. Mean with S.E.M. is reported, p-value on graph is from two-tailed t-test.

## Description

*sym-2* encodes an ortholog of the RNA-binding proteins hnRNPF and hnRNPH. A putative loss-of-function allele, *sym-2(mn617)* from(Davies *et al.*, 1999) is a Y163N missense mutation, which lies N-terminal to the first RNA-recognition motif of SYM-2. Homozygous animals have no overt defects but animals heterozygous for *sym-2(mn617)* and homozygous for *mec-8* mutations are embryonic lethal (Davies *et al.*, 1999; Yochem *et al.*, 2004). Additionally, *sym-2(mn617)* suppresses the exhaustion-induced locomotion defect of *smn-1(cb131)* animals (Walsh *et al.*, 2020). We have previously shown that human hnRNPA2, an ortholog of the protein encoded by *C. elegans hrpa-1*, interacts with hnRNPF *in vitro* (Ryan *et al.*, 2020). As the low complexity (LC) domains of hnRNPA2 and HRPA-1 are not well conserved, we replaced the third coding exon of the *C. elegans hrpa-1* gene with the corresponding human protein sequence codon optimized for *C. elegans* expression, resulting in a chimeric HRPA-1 protein with the human LC domain (HRPA-1HsLC^WT^) (Ryan *et al.*, 2020). A mutation in hnRNPA2, D290V, is associated with multisystem proteinopathy, a disease that causes degeneration of muscles, bone, and neurons (Kim *et al.*, 2013). Expression of the mutant chimeric version of *hrpa-1*, *hrpa-1*HsLC^D290V^, causes glutamatergic neurodegeneration in phasmid neurons after oxidative stress in animals lacking endogenous *hrpa-1* function (Ryan *et al.*, 2020). To control for defects associated with array integration, we also created an empty array, *hrpa-1*HsLC^empty^, which contains no *hrpa-1*. We hypothesized that *sym-2(mn617)* might rescue *hrpa-1*HsLC^D290V^ stress-induced neurodegeneration.

To test this hypothesis, we generated animals carrying *sym-2(mn617)* with *hrpa-1*(*tm781*) and *hrpa-1*HsLC variants. We first exposed all genotypes to 22 hours of paraquat-induced oxidative stress and counted intact neurons after dye filling. We found that *sym-2(mn617)* rescued *hrpa-1*HsLC^D290V^ stress-induced neurodegeneration but we also found that about 25% of the neurons in *sym-2(mn617)* animals failed to dye fill after 22 hours of stress. Virtually all *sym-2(mn617)* neurons dye filled after only 4 hours of stress; therefore, the dye filling defect is degeneration induced by stress. Interestingly, we found that *sym-2(mn617)* neurodegeneration is sensitive to *hrpa-1* function. Animals expressing both *sym-2(mn617)* and *hrpa-1*HsLC^WT^ showed significant rescue of the *sym-2(mn617)* defect back to WT levels. *hrpa-1(tm781)*, *hrpa-1*HsLC^D290V^, *hrpa-1*HsLC^empty^ did not rescue the *sym-2(mn617)* defect.

The identification of neurodegeneration in *sym-2(mn617)* animals after longer exposure to oxidative stress was surprising, especially given the suppression of neuromuscular defects in *smn-1(cb131)* (Walsh *et al.*, 2020) and improvement of *hrpa-1*HsLC^D290V^ defects. However, HNRNPF, an ortholog of *sym-2*, is associated with the neurodevelopmental disorder Rett syndrome (Newnham *et al.*, 2010), suggesting that HNRNPF has an important role in preserving neuronal integrity. Interestingly, although *hrpa-1*HsLC^WT^ can rescue the *sym-2(mn617)* neurodegeneration, *hrpa-1*HsLC^D290V^ has similar levels of neurodegeneration as *sym-2(mn617)*, with substantially less neurodegeneration than observed for *hrpa-1*HsLC^D290V^ alone. As such, *sym-2* may be in a genetic pathway upstream of *hrpa-1*, although more work is needed to elucidate what this pathway is and how it leads to neurodegeneration.

## Methods

Glutamatergic neurodegeneration: Day 1 adult animals were washed off plates with M9 and incubated with DiD (Fisher DilC18(5) D307) in a microfuge tube as in (Perkins *et al.*, 1986). After 1.5 hours, animals were spun down at 10000 rpm for 30 seconds and transferred to a regular NGM plate. After 30 minutes, animals were mounted on 2% (vol/vol) agar pads and immobilized in 30 mg/mL 2-3-butaneione monoxime (BDM, Sigma) in M9 buffer. Fluorescent neuronal cell bodies were visualized and scored for dye uptake under 63x objectives. There are 4 phasmid neurons per animal, two per side. Neurons were scored as intact if the cell body took up fluorescent dye. For trials with paraquat stress, animals were exposed to 2.5 mM paraquat on plates for the indicated time frame.

Statistical analysis: Data collection and analysis were performed by experimenters blinded to genotype. Quantitative data were analyzed using GraphPad Prism 7. Two tailed t-test was used to determine significance for neurodegeneration assays. A value of P < 0.05 was used to establish statistical significance. Error bars in figures represent error of the mean (S.E.M.). A one-way ANOVA was performed with a Tukey post-hoc test for the 55 comparisons; these corrected p values are presented in the figure legend.

## Reagents

*C. elegans* were maintained on Nematode Growth Media (NGM) that was seeded with *E. coli* strain OP50 as a food source according to established protocols (Brenner, 1974). Animals were maintained at 20˚C. All animals except *sym-2(mn617*) and N2 were maintained over the *tmC25[tmIs1241]* balancer. Experimenter was blinded to genotype for all trials and all trials were independent.

The *sym-2* mutant strains described herein are available by request (anne_hart@brown.edu). They are:

**Table:** 

HA3578	*sym-2(mn617) II*
HA3610	*hrpa-1(tm781)/tmC25[tmIs1241] IV; sym-2(mn617) II*
HA3766	*rtIs83[hrpa-1p::hrpa-1HsLC^WT^::hrpa-1 3’UTR + elt-2p::GFP + salmon sperm DNA]; hrpa-1(tm781)/tmC25[tmIs1241] IV; sym-2(mn617) II*
HA3768	*rtIs82[hrpa-1p::hrpa-1HsLC^D290V2^::hrpa-1 3’UTR + elt-2p::GFP + salmon sperm DNA]; hrpa-1(tm781)/tmC25[tmIs1241] IV; sym-2(mn617) II*
HA3769	*rtIs93[hrpa-1p::hrpa-1HsLC^empty1^::hrpa-1 3’UTR + elt-2::GFP + salmon sperm DNA]; hrpa-1(tm781)/tmC25[tmIs1241] IV; sym-2(mn617) II*

The following previously published strains were used in this study:

N2 Wild type, Bristol, (Brenner, 1974)

From (Ryan *et al.*, 2020):

**Table:** 

HA3608	*tmC25[tmIs1241]/+ IV*
HA3450	*hrpa-1(tm781)/tmC25[tmIs1241] IV*
HA3655	*rtIs83[hrpa-1p::hrpa-1HsLC^WT^::hrpa-1 3’UTR + elt-2p::GFP + salmon sperm DNA]; hrpa-1(tm781)/tmC25[tmIs1241] IV*
HA3659	*rtIs82[hrpa-1p::hrpa-1HsLC^D290V2^::hrpa-1 3’UTR + elt-2p::GFP + salmon sperm DNA]; hrpa-1(tm781)/tmC25[tmIs1241] IV*
HA3708	*rtIs93[hrpa-1p::hrpa-1HsLC^empty1^::hrpa-1 3’UTR + elt-2::GFP + salmon sperm DNA]; hrpa-1(tm781)/tmC25[tmIs1241] IV*

All strains used here are available by request (anne_hart@brown.edu).
